# 920. A Phase 2 Multi-Center, Prospective, Randomized, Double-Blind Study to Assess the Clinical and Antiviral Efficacy and Safety of Nitazoxanide for the Treatment of Norovirus in Hematopoietic Stem Cell and Solid Organ Transplant Recipients

**DOI:** 10.1093/ofid/ofad500.965

**Published:** 2023-11-27

**Authors:** Catherine-Audrey Boutin, Michelle A Callegari, Diana F Florescu, Minh-Hong Nguyen, Daniel Kaul, Robin K Avery, Pearlie P Chong, Cynthia E Fisher, Ajit Limaye, Lisa A Clough, Steven A Pergam, Michael D Green, Marian G Michaels, Lara A Danziger-Isakov, Michael P Angarone, Laurie Keefer, Amna Daud, Michael Ison

**Affiliations:** CHUM, Université de Montréal, Montréal, Quebec, Canada; Northwestern University, Chicago, Illinois; University of Nebraska Medical Center, Omaha, Nebraska; University of Pittsburgh, Pittsburg, Pennsylvania; University of Michigan, Ann Arbor, Michigan; Johns Hopkins, Baltimore, Maryland; University of Texas Southwestern, Dallas, TX; University of Washington, Seattle, Washington; university of washington, Seattle, Washington; The University of Kansas Medical Center, Kansas City, Kansas; Fred Hutchinson Cancer Research Center; University of Washington, Seattle, WA; University of Pittsburgh School of Medicine, Pittsburgh, PA; UPMC Children's Hospital of Pittsburgh, Pittsburgh, Pennsylvania; Cincinnati Children's Hospital, Cincinnati, Ohio; Northwestern University Feinberg School of Medicine, Chicago, IL; Mount Sinai, New York, New York; Northwestern University Feinberg School of Medicine, Chicago, IL; Respiratory Diseases Branch, DMID/NIAID/NIH, Derwood, MD

## Abstract

**Background:**

Norovirus (NoV) results in potentially severe, relapsing, remitting diarrhea in immunocompromised hosts (ICH). A number of interventions, including nitazoxanide (NTZ), have been tried with unclear success in managing cases of NoV in ICH.

**Methods:**

We conducted a NIH-sponsored multi-center, prospective, randomized, double-blind study of NTZ for the treatment of Norovirus in adult HSCT and SOT recipients between 2018 and 2021. Subjects with a positive Norovirus test within 14 days of enrollment and active GI symptoms were randomly assigned (1:1) to NTZ 500 mg twice daily or placebo (P) for 56 consecutives doses and were followed for 6 months, including patient reported outcomes (PRO) diary assessments. Primary endpoint was to determine the clinical efficacy, assessed as the time from randomization until symptoms resolution for at least 48 hours. Secondary endpoints included virologic efficacy assessed as the time from randomization to first negative viral load and safety through frequency of adverse events.

**Results:**

31 subjects (16 NTZ, 15 P) were enrolled and had balanced demographics (See Table 1). Early withdrawal was documented in 5 subjects from each group. Thirty (30) had received solid organ transplants. Most had chronic ( > 14 days) symptoms (77%). In the mITT population, the median time to initial clinical resolution was 19.0 days (95% CI: 1.0, 31.0) for the Nitazoxanide group and 11.0 days (95% CI: 2.0, 14.0) for the placebo group (p-value=0.459). The difference between time to first negative viral load for the Norovirus GII genotype was not significant (p-value=0.873). Patients appear to have clinical improvement based on PRO results while on active therapy. No serious adverse event related to the study treatment was documented. One severe unsolicited adverse event, abdominal pain, was reported on the day of first dose NTZ. Hospitalization and non-serious or laboratory adverse events were not significantly different between the two arms. Analysis of PK and viral genetics is ongoing and will be reported at the meeting.

**Figure:**
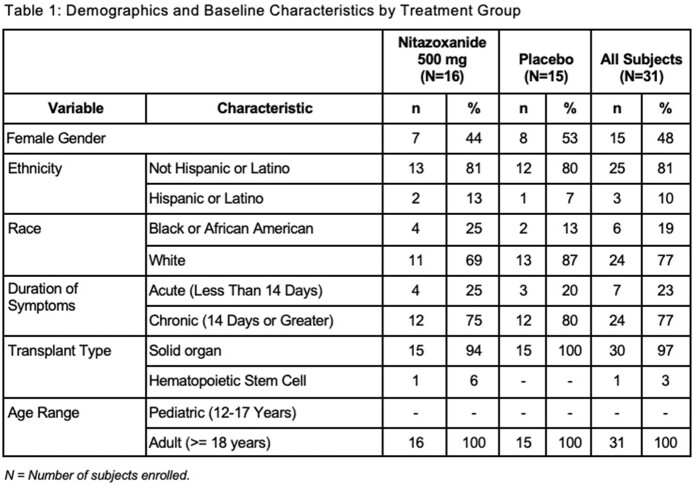

**Figure:**
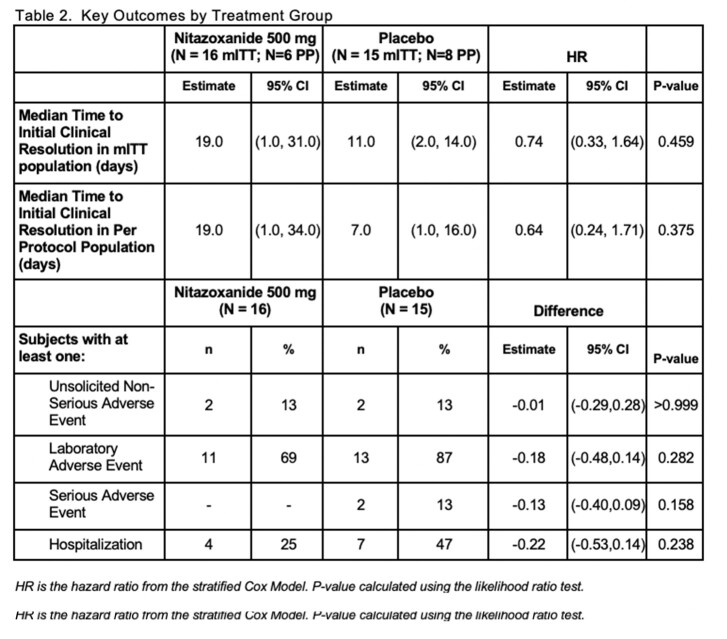

**Figure:**
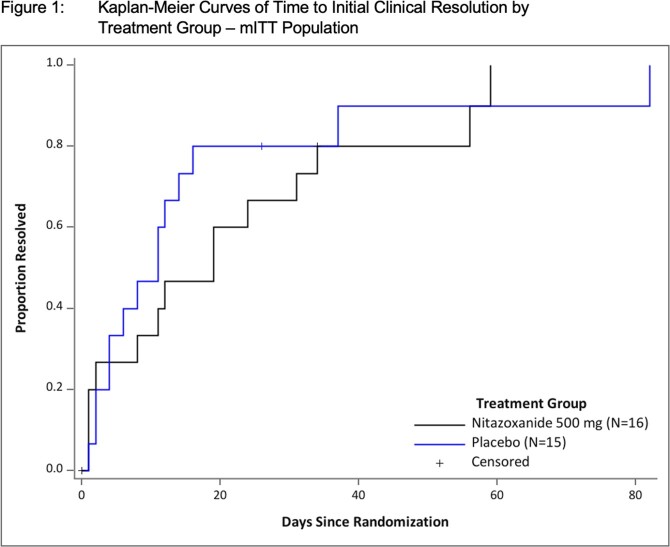

**Conclusion:**

NTZ did not shorten time to clinical resolution or viral shedding duration but may have resulted in transient symptom improvement. Although NTZ appears safe, its role is likely limited in the setting of chronic NoV among ICHs.

**Disclosures:**

**Daniel Kaul, MD**, Medscape: Honoraria|Nobelpharma: Grant/Research Support|Takeda: Grant/Research Support **Robin K. Avery, MD**, Aicuris: Grant/Research Support|Astellas: Grant/Research Support|Astra-Zeneca: Grant/Research Support|Chimerix: Grant/Research Support|Merck: Grant/Research Support|Oxford Immunotec: Grant/Research Support|Qiagen: Grant/Research Support|Regeneron: Grant/Research Support|Takeda: Grant/Research Support **Ajit Limaye, Professor/MD**, MedPace: DSMB member|merck: Advisor/Consultant|merck: Grant/Research Support|moderna: Advisor/Consultant|moderna: site investigator|syneos: DSMB member **Steven A. Pergam, MD, MPH**, Cidara: Investigator in clinical trials|F2G: Investigator in clinical trials|Global Life Technologies: Grant/Research Support|Symbio: Investigator in clinical trials **Michael D. Green, MD, MPH**, ADMA: Advisor/Consultant|Allovir: Advisor/Consultant|Bristol Myers Squibb: Advisor/Consultant|ITB-MED: Advisor/Consultant **Marian G. Michaels, MD, MPH**, Merck: Grant/Research Support|Viracor: Grant/Research Support **Lara A. Danziger-Isakov, MD, MPH**, Aicuris: Contracted Clinical Research|Ansun Biopharma: Contracted Clinical Research|Astellas: Contracted Clinical Research|GSK: Advisor/Consultant|Merck: Advisor/Consultant|Merck: Contracted Clinical Research|Pfizer: Contracted Clinical Research|Roche Diagnostics: Advisor/Consultant|Takeda: Advisor/Consultant|Takeda: Contracted Clinical Research **Michael P. Angarone, DO**, Abbvie Pharmeciuticals: Advisor/Consultant|DKBMed Inc: Advisor/Consultant|DKBMed Inc: Honoraria

